# Early-life cow-level risk factors for sole ulcers in primiparous dairy cows

**DOI:** 10.3168/jdsc.2024-0544

**Published:** 2024-05-10

**Authors:** Peter T. Thomsen, Hans Houe

**Affiliations:** 1 Department of Animal and Veterinary Sciences, Aarhus University, DK-8830 Tjele, Denmark; 2 Department of Veterinary and Animal Sciences, University of Copenhagen, DK-1870 Frederiksberg C, Denmark

## Abstract

Sole ulcers negatively affect cow welfare and production economy. Previous cases of sole ulcers increase the risk of new or recurrent cases, and prevention of sole ulcers during the first lactation may therefore have a long-term effect, also in later lactations. Until now, risk factors for sole ulcers in the first lactation, associated with the period before heifers calve for the first time, have not been investigated. This study evaluated early-life cow-level risk factors for sole ulcers at the first hoof trimming in the first lactation. Early-life risk factors were defined as risk factors associated with the period before a cow calves for the first time. A dataset including information about all hoof trimming recordings during the years 2020 to 2022, and information about individual cows, was retrieved from the Danish Cattle Database. The dataset included only primiparous cows, and only cows that were hoof trimmed at least once during the first lactation. Overall, 1.95% of 466,113 cows included in the study had sole ulcers at the first hoof trimming in the first lactation. Results from a logistic regression model demonstrated a statistically significant interaction between breed and age at first calving (grouped based on quartiles within breed). Overall, across breeds, odds ratios of sole ulcers at the first hoof trimming in the first lactation were generally approximately 0.5 in the first quartile of age at first calving, approximately 0.6 in the second quartile, and approximately 0.75 in the third quartile, compared with the fourth quartile within the same breed. Odds of sole ulcers were generally higher in Jersey and Danish Red Dairy cows, compared with Holstein. In conclusion, the odds of sole ulcers in the first lactation increased with increasing age at first calving, and was approximately twice as high in cows calving among the oldest 25% within a breed, compared with cows calving among the youngest 25%. Focus on early-life risk factors for sole ulcers may have a major influence on the occurrence of sole ulcers throughout the life of cows.

## Highlights

• Previous cases of sole ulcers increase the risk of new cases.
• Prevention of sole ulcers in the first lactation may thus have long-term effects.
• Overall, 1.95% of primiparous cows had sole ulcers.
• Odds of sole ulcers increased with increasing age at first calving.
• Cows calving among the oldest 25% had twice as high odds as the youngest 25%.

## Graphical Abstract

**Figure:**
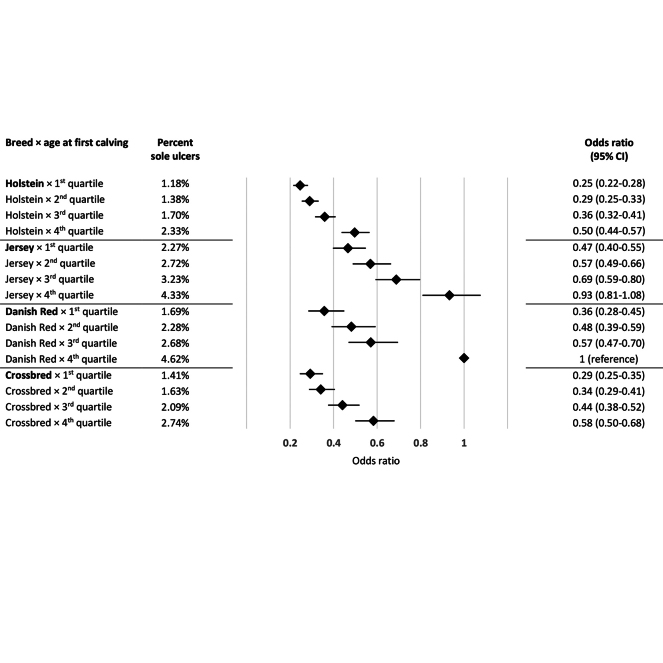
**Summary:** Previous cases of sole ulcers increase the risk of new cases, and prevention of sole ulcers during the first lactation may, therefore, have long-term effects. This study evaluated early-life risk factors (associated with the period before first calving) for sole ulcers at the first hoof trimming in the first lactation. Analysis of data from 466,113 primiparous Danish dairy cows from 2020 to 2022 showed that 1.95% of cows had sole ulcers at the first hoof trimming in the first lactation. Results from a logistic regression model showed that the odds of sole ulcers were approximately twice as high in cows calving for the first time among the oldest 25% within a breed, compared with cows calving among the youngest 25%. Focus on early-life risk factors for sole ulcers may influence the occurrence of sole ulcers throughout the life of cows.

Lameness is a major problem in modern dairy production and has negative effects on milk yield, production economy, and animal welfare (Thomsen et al., 2023). Most cases of lameness in dairy cows are caused by hoof lesions (Murray et al., 1996). Several studies have, based on the cost per case, estimated that sole ulcers are the most costly hoof lesion in dairy cows (Cha et al., 2010; Charfeddine and Pérez-Cabal, 2017; Dolecheck et al., 2019). Sole ulcers generally affect cows for a long time and likely cause prolonged pain (Pirkkalainen et al., 2022). Hoof horn lesions, including sole ulcers, and especially chronic cases, are difficult to treat (Thomas et al., 2016), and therefore prevention should be a key focus when it comes to the management of sole ulcers in dairy cows. Risk factors for sole ulcers have been identified in dairy cows (e.g., Holzhauer et al., 2008; Thomsen et al., 2019). However, to our knowledge, risk factors associated with the period before heifers calve for the first time have never been investigated before. It has been shown that previous cases of sole ulcers increase the risk of new or recurrent cases. Thomsen et al. (2019) generally found 3 times higher odds of sole ulcers in cows with sole ulcers in the previous lactation. Oikonomou et al. (2013) generally found approximately twice as high a risk of sole ulcers in cows having had sole ulcers in a previous lactation. As there seems to be a carry-over effect from previous lactations, prevention of sole ulcers in the first lactation is likely to have a positive effect, not only during the first lactation, but also during subsequent lactations. This possible long-term effect of prevention of sole ulcers in the first lactation makes the evaluation of risk factors for sole ulcers in the first lactation especially interesting.

Since 2009, Danish farmers, veterinarians, and hoof trimmers have been able to record hoof lesions and report them digitally to the Danish Cattle Database. The vast majority of recordings are made by hoof trimmers during routine hoof trimmings. In 2022, approximately 761,000 recordings were made among the approximately 556,000 dairy cows in Denmark. More than half of these recordings (∼419,000) were recordings of hoof trimmings without any lesions recorded, and in approximately 342,000 cases one or more hoof lesions were recorded (Capion et al., 2021; Landbrugsinfo, 2023). Several studies have tested the accuracy of the recordings made by hoof trimmers against an independent observer. The accuracy of the recordings is variable, but has been shown to be highest for digital dermatitis, interdigital hyperplasia, and sole ulcers (Capion et al., 2021).

The objective of this study was to evaluate early-life cow-level risk factors for sole ulcers at the first hoof trimming in the first lactation. Early-life risk factors were defined as risk factors associated with the period before a cow calves for the first time, and thus include factors associated with the period from the birth of the cow until she calves for the first time.

The Danish Cattle Database is managed by the Danish Cattle Federation (Skejby, Denmark) and includes information on individual animals about, for example, disease treatments, milk yield and composition, pedigree, hoof trimmings, movements of animals, births, and deaths. Information is collected from farmers, veterinarians, hoof trimmers, slaughterhouses, laboratories, cattle breeding organizations, and so on. In July 2023, a dataset including information about all hoof trimming recordings during the years 2020 to 2022 was retrieved from the Danish Cattle Database. Additionally, the dataset included information about each cow (e.g., date of birth, size at birth, disease treatments, and calving dates). The dataset included only primiparous cows, and only cows that were hoof trimmed at least once during the first lactation. In cases where cows were trimmed more than once during the first lactation, only information about the first hoof trimming was included. Hoof trimmings done more than 500 d after the first calving were not included. Cows calving for the first time at an age of younger than 500 d (∼16 mo) or older than 1,000 d (∼33 mo) were not included. These cows constituted approximately 0.6% of the cows in the original dataset. To exclude any effect of preexisting sole ulcer lesions, cows with a sole ulcer diagnosed before the first calving were excluded from the dataset. The final dataset included information about the first hoof trimming in the first lactation from 466,113 cows from 1,748 different herds.

For the analyses, several explanatory variables were extracted or calculated from the dataset. The variables are presented in Table 1 together with descriptive statistics. Except for hoof trimmings, recording of all variables in Table 1 is mandatory for Danish farmers and veterinarians. Dystocia refers to the birth of the cow—not her own first calving. Dystocia is recorded by Danish dairy farmers for all calvings and was grouped as yes (difficult calving with help from farmer or veterinarian, or cesarean section) or no. Likewise, farmers report the size of the calf recorded as small or large (no middle category), and whether the calf was born as a twin (or triplet) or not. All disease treatments done by veterinarians and farmers must be reported to the Danish Cattle Database. Based on these recordings, it was recorded whether the cow had been treated for diarrhea or pneumonia at least once before her first calving. Based on information about dates of hoof trimmings and first calving, cows were grouped as not being trimmed before first calving, trimmed early (more than 90 d before first calving), median (90 to 31 d before first calving), or late (30 d or less before first calving). Cows were grouped in relation to age at first calving based on quartiles. As the age at first calving differed between breeds, this grouping was done for each breed separately. The median age at first calving was 738 d for Holstein, 699 d for Jersey, 749 d for Danish Red Dairy, and 738 d for crossbred cows. Table 1 presents the quartile thresholds for each breed.

**Table 1:** Descriptive statistics of explanatory variables from an analysis of the association with sole ulcers at the first hoof trimming in the first lactation

Variable	Levels	Proportion in each level (%)	Proportion with sole ulcer (%)
Breed	Holstein	68.3	1.65
	Jersey	13.3	3.14
	Danish Red Dairy	5.2	2.82
	Crossbred	13.2	1.97
Dystocia	No	99.4	1.96
	Yes	0.6	2.16
Size at birth	Small	42.3	1.99
	Large	57.7	1.97
Born as twin	No	97.6	1.95
	Yes	2.4^1^	1.84
Treated for diarrhea before first calving	No	93.3	1.96
	Yes	6.7	1.83
Treated for pneumonia before first calving	No	85.4	1.95
	Yes	14.6	1.96
Hoof trimming before first calving	No	69.8	1.97
	Early (>90 d)	9.0	1.98
	Median (90–31 d)	11.9	1.94
	Late (≤30 d)	9.3	1.81
Quartile of age at first calving by breed	Holstein, first quartile (<706 d)	24.6	1.18
	Holstein, second quartile (706–737 d)	25.1	1.38
	Holstein, third quartile (738–779 d)	25.2	1.70
	Holstein, fourth quartile (>779 d)	25.1	2.33
	Jersey, first quartile (<667 d)	24.8	2.27
	Jersey, second quartile (667–698 d)	24.9	2.72
	Jersey, third quartile (699–741 d)	25.3	3.23
	Jersey, fourth quartile (>741 d)	25.1	4.33
	Danish Red, first quartile (<716 d)	25.3	1.69
	Danish Red, second quartile (716–748 d)	24.9	2.28
	Danish Red, third quartile (749–792 d)	25.0	2.68
	Danish Red, fourth quartile (>792 d)	24.9	4.62
	Crossbred, first quartile (<705 d)	24.9	1.41
	Crossbred, second quartile (705–737 d)	25.1	1.63
	Crossbred, third quartile (738–779 d)	24.9	2.09
	Crossbred, fourth quartile (>779 d)	25.1	2.74

1 0.02% were triplets.

All explanatory variables were included in a logistic regression model (PROC GLIMMIX, SAS 9.3; SAS Institute Inc.) with sole ulcer at the first hoof trimming in the first lactation (yes/no) as the outcome. Stage of lactation has previously been shown to be a risk factor for sole ulcers in dairy cows. The risk or odds of sole ulcers is typically highest 4 to 6 mo after calving (Solano et al., 2016; Thomsen et al., 2019). Even though it is not an explanatory variable associated with the period before the first calving, month in milk was therefore included in the logistic regression model as a covariate. Month in milk was coded as 1 (0–30 DIM, 19.3% of all observations), 2 (31–60 d, 16.7%), 3 (61–90 d, 20.0%), 4 (91–120 d, 15.4%), 5 (121–150 d, 8.2%), 6 (151–180 d, 4.9%), or 7+ (>180 d, 15.6%). To account for clustering of cows within herds, herd was included as a random effect. The model was reduced using backward elimination with explanatory variables with *P* < 0.05 removed from the model. Possible interactions between statistically significant explanatory variables were included in the final model. Goodness-of-fit was assessed using the dispersion parameter (Pearson χ^2^/df), which should be close to 1.

Overall, 1.95% of the 466,113 cows had sole ulcers at the first hoof trimming in the first lactation. Figure 1 illustrates the temporal distribution according to month in milk of sole ulcer diagnoses. Sole ulcers were diagnosed at a median of 81 DIM (mean: 103) with only minor differences between breeds: median DIM at diagnosis were 79, 87, 78, and 84, for Holstein, Jersey, Danish Red, and crossbred cows, respectively. Table 1 presents the proportion of sole ulcers for each level of the explanatory variables. The dispersion parameter was 1.01, indicating acceptable fit of the logistic regression model. Breed and age group were both statistically significant, and we found a statistically significant interaction between them (*P* < 0.0001). Results from the logistic regression are presented in Figure 2. Overall, across Holstein, Jersey, and crossbred cows, odds ratios of sole ulcers were generally approximately 0.5 in the first quartile of age at first calving, approximately 0.6 in the second quartile, and approximately 0.75 in the third quartile, compared with the fourth quartile. The same overall trend was seen for Red Danish Dairy cows. However, here the proportions between odds for the quartiles were slightly different with odds ratios of approximately 0.35, 0.5, and 0.6 in the first, second, and third quartiles, respectively, compared with the fourth quartile.

**Figure 1:**
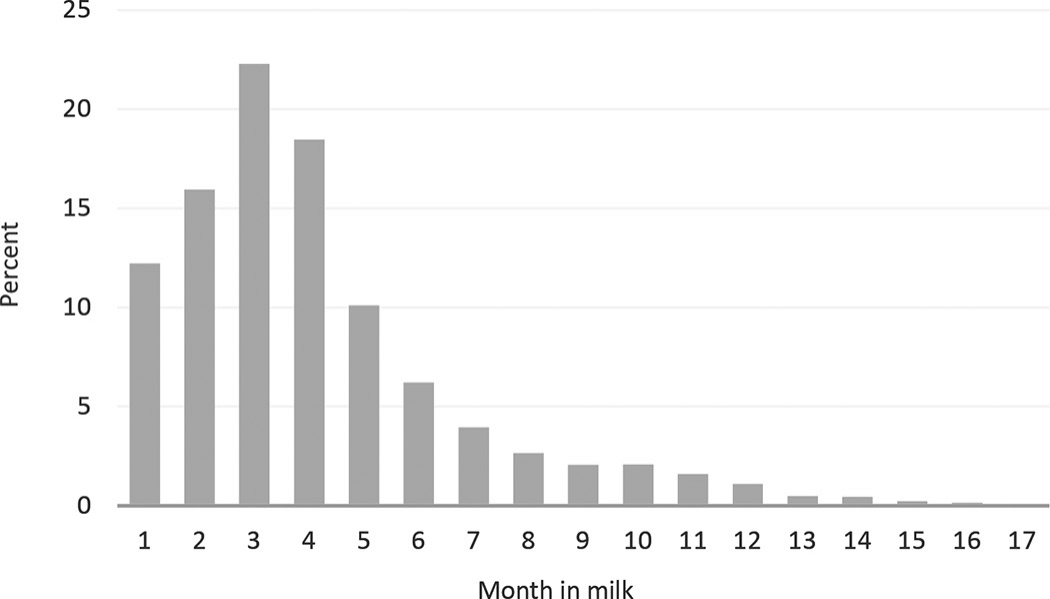
Temporal distribution according to month in milk of sole ulcer diagnoses in 466,113 primiparous Danish dairy cows.

**Figure 2:**
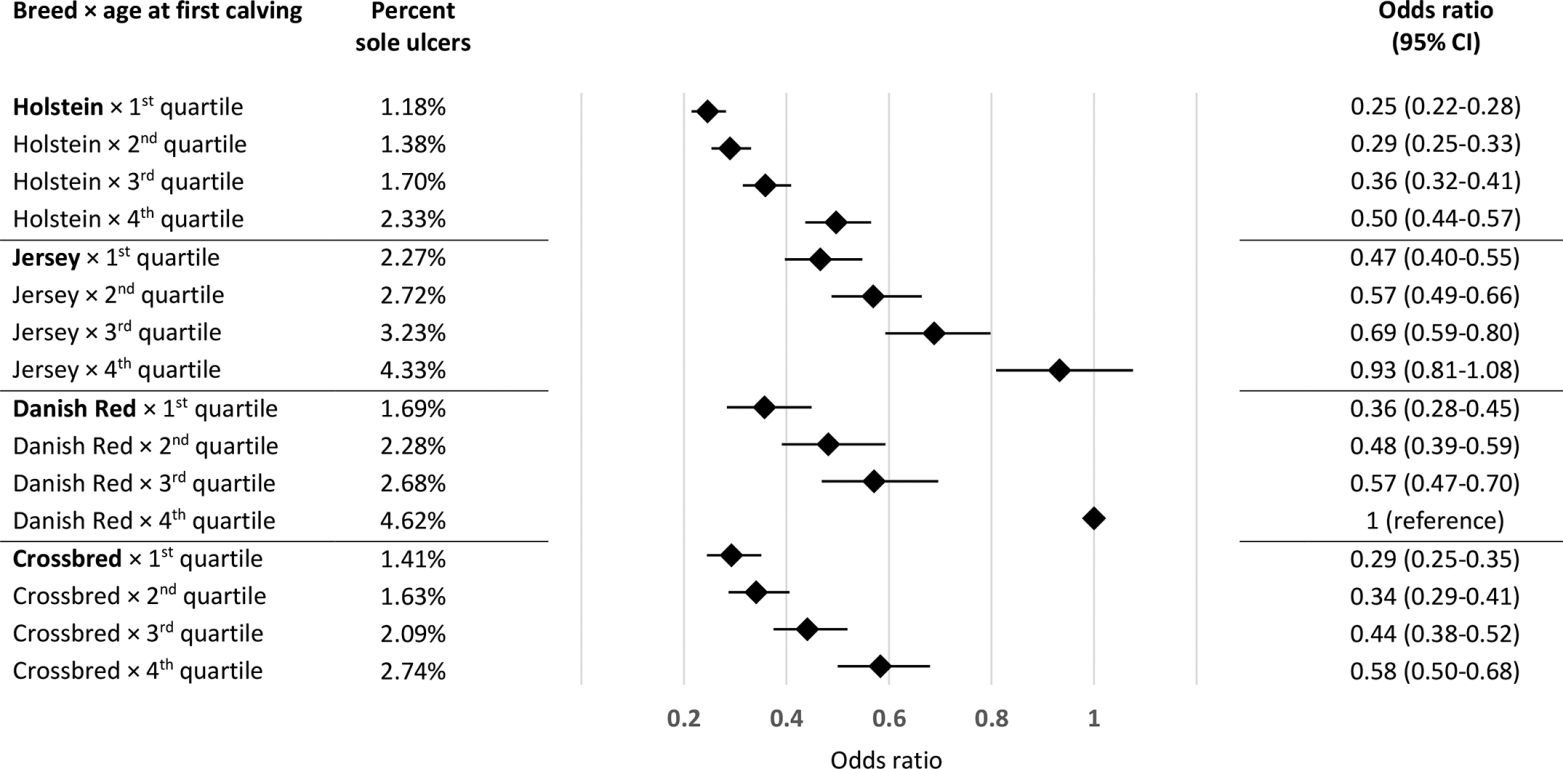
Percentage of cows within group with sole ulcers and odds ratios from a logistic regression model evaluating odds of sole ulcers at the first hoof trimming in the first lactation in 466,113 Danish dairy cows. Rhombi represent odds ratios, and horizontal lines indicate 95% CI. The interaction between breed and age at first calving (based on quartiles within breed) was highly significant (*P* < 0.0001).

Age at first calving had a statistically significant association with the odds of sole ulcers at the first hoof trimming in the first lactation. Cows calving among the oldest 25% within a breed had approximately twice as high odds of sole ulcers as cows calving among the 25% youngest. Differences in age at first calving were relatively minor: Across breeds, the difference between the youngest and oldest quartile was a little more than 2 mo (73–76 d, Table 1). In contrast to many of the other potential risk factors associated with the early life of a cow, age at first calving is an example of a manageable risk factor. A manageable risk factor can be defined as a risk factor that can be changed, eliminated, or mitigated without too much extra work, expenses, or unwanted side effects. Most farmers are able to change the mean age at first calving in their herd without too much extra work or costs. In contrast, other potential risk factors may be extremely difficult—or impossible—for farmers to change. As an example, even if being born as a twin had been a risk factor for sole ulcers in the first lactation, farmers would have had very limited possibility to change this risk factor.

Previous research has demonstrated that hoof trimming around drying off may help reduce the odds of sole ulcers in the following lactation (Thomsen et al., 2019). In contrast to this, we found no evidence of an effect of hoof trimming heifers before their first calving on the odds of sole ulcers in the first lactation. Heifers may be hoof trimmed before the first calving either due to a (suspected) specific hoof pathology or as a routine preventive procedure. In the first case, the odds of sole ulcers in the first lactation may be increased due to hoof lesions already existing at calving. In the latter case, a positive effect on hoof health may be expected. It is possible that these 2 mechanisms counteract each other and result in no effect of hoof trimming in heifers overall. In the present study, we excluded cows with sole ulcers diagnosed before the first calving, but it is possible that other hoof horn lesions, or hoof trimmings correcting abnormal hoof conformation, may still affect the risk of sole ulcers after calving. Research on the potential benefits of hoof trimming in dairy heifers is sparse (Mahendran and Bell, 2015) and should be prioritized in the future.

The etiology behind lower odds of sole ulcers in cows calving for the first time at a younger age remains unclear. However, several mechanisms may be involved, independently or in combination. Dairy heifers typically have an average daily weight gain of close to 1 kg per day in late gestation (PennState Extension, 2022). Therefore, all other things equal, a heifer being, for example, 27 mo old when calving for the first time will be almost 100 kg heavier than a heifer calving at 24 mo old. Pressure on the sole has been suggested as part of the etiology of sole ulcers (Ouweltjes et al., 2019), and it is possible that a higher BW at first calving will increase the risk of sole ulcers during the first lactation, thus explaining the association between age at first calving and the odds of sole ulcers. The mean age at first calving has been termed a “key performance parameter” in dairy herds (Steele, 2020, p. 2), and has been described as “one of the most frequently used parameters to evaluate the efficacy of replacement rearing programs in dairy herds” (Vargas-Leitón et al., 2023, p. 1). It is possible that achieving a relatively low mean age at first calving is an indicator of a highly qualified and skilled farmer. Such farmers may also do many other things correct, including measures reducing the risk of sole ulcers in their cows. In other words, the “good” farmer able to obtain a low mean age at first calving may also be good at preventing sole ulcers.

Results from the present study have shown that a younger age at first calving is associated with lower odds of sole ulcers in the first lactation. However, advising farmers about the optimal age at first calving is not straightforward. The effect of age at first calving on other health and production parameters needs to be taken into account. Age at first calving has been shown to be associated with, for example, survival, risk of dystocia, production economy, fertility, and milk production (Ettema and Santos, 2004; Sherwin et al., 2016; Eastham et al., 2018; Atashi et al., 2021). It is important to find a balance between optimal prevention of sole ulcers and other potential effects of age at first calving. In particular, it is important to avoid any negative side effects of a young age at first calving.

We found an association between breed and sole ulcers, with higher odds of sole ulcers at the first hoof trimming in the first lactation in Jersey and Danish Red Dairy cows. The association between breed and prevalence, risk, or odds of sole ulcers has been evaluated in a few previous studies. However, none of these other studies have focused only on primiparous cows. Kujala et al. (2009) found an almost 3 times higher risk of sole ulcers in Finnish Holstein cows, compared with Finnish Ayrshire. Studying only second parity and older cows, Thomsen et al. (2019) found relatively small breed differences, with the lowest odds of sole ulcers in Jersey and crossbred cows. Arango-Sabogal et al. (2020) found odds of sole ulcers in Holstein cows from Québec to be approximately twice as high as in other breeds. In contrast, Capion et al. (2021) found only minor breed differences in the prevalence of sole ulcers in Danish dairy cows hoof trimmed during 2013 to 2017.

In conclusion, this study has demonstrated that odds of sole ulcers at the first hoof trimming in the first lactation is higher in cows calving for the first time at an older age. The odds of sole ulcers was approximately twice as high in cows calving among the oldest 25% within a breed, compared with cows calving among the youngest 25%. As cows having experienced previous episodes of sole ulcers have an increased risk of new or recurrent cases in subsequent lactations, prevention of sole ulcers in the first lactation may have a long-term effect. Focus on early-life risk factors for sole ulcers may therefore have a major influence on the occurrence of sole ulcers throughout the life of cows, even though the prevalence of sole ulcers is generally lower in the first lactation than in later lactations.
